# Low-Attenuation Coronary Plaque Volume and Cardiovascular Events in Patients with Distinct Metabolic Phenotypes with or without Diabetes

**DOI:** 10.31083/j.rcm2412361

**Published:** 2023-12-25

**Authors:** Kenichiro Otsuka, Hirotoshi Ishikawa, Kenei Shimada, Kana Hojo, Hiroki Yamaura, Yasushi Kono, Noriaki Kasayuki, Daiju Fukuda

**Affiliations:** ^1^Department of Cardiovascular Medicine, Osaka Metropolitan University Graduate School of Medicine, 545-8585 Osaka, Japan; ^2^Department of Cardiovascular Medicine, Fujiikai Kashibaseiki Hospital, 639-0252 Kashiba, Japan

**Keywords:** acute coronary syndrome, adipose tissue, computed tomography angiography, coronary artery disease, diabetes mellitus

## Abstract

**Background:**

Diabetes mellitus (DM) plays a key role in the pathophysiology of metabolic syndrome (MetS). This study aimed to investigate the association among DM, low-attenuation plaque (LAP) volume, and cardiovascular outcomes across metabolic phenotypes in patients with suspected coronary artery disease (CAD) who underwent coronary computed tomography angiography (CCTA).

**Methods:**

We included 530 patients who underwent CCTA. MetS was defined as the presence of a visceral adipose tissue area ≥100 ${\mathrm{cm}}^{2}$ in patients with DM (n = 58) or two or more MetS components excluding DM (n = 114). The remaining patients were categorised as non-MetS patients with DM (n = 52) or without DM (n = 306). A CCTA-based high-risk plaque was defined as a LAP volume of >4%. The primary endpoint was the presence of a major cardiovascular event (MACE), which was defined as a composite of cardiovascular death, acute coronary syndrome, and coronary revascularization.

**Results:**

The incidence of MACE was the highest in the non-MetS with DM group, followed hierarchically by the MetS with DM, MetS without DM, and non-MetS without DM groups. In the multivariable Cox hazard model analysis, DM as a predictor was associated with MACE independent of LAP volume >4% (hazard ratio, 2.68; 95% confidence interval, 1.16–6.18; *p* = 0.02), although MetS did not function as an independent predictor. A LAP volume >4% functioned as a predictor of MACE, independent of each metabolic phenotype or DM.

**Conclusions:**

This study demonstrated that DM, rather than MetS, is a predictor of coronary events independent of high-risk plaque volume in patients who underwent CCTA.

## 1. Introduction

The global prevalence of diabetes mellitus (DM) and metabolic syndrome (MetS) has increased significantly over the past decades, contributing to an increased risk of atherosclerotic cardiovascular disease (ASCVD) [1]. Clinical studies have demonstrated that being overweight (25–29.9 kg/$m^{2}$) or obese (≥30 kg/$m^{2}$), as defined by the body mass index (BMI) alone, reflects heterogeneous body fat distribution and distinct metabolic conditions [2]. This observation raises questions regarding the relationship between BMI and ASCVD risk, which leads to the obesity paradox [3, 4, 5]. Although visceral adiposity, a modifiable risk factor for the development of MetS, helps to identify metabolically unhealthy individuals [1, 2, 6, 7], the coronary plaque features associated with ASCVD events in individuals with distinct metabolic phenotypes remain largely unknown.

Coronary computed tomography angiography (CCTA) facilitates the diagnosis of coronary artery disease (CAD) and offers a prognostic value based on high-risk coronary plaque features beyond stenosis severity [8]. Furthermore, a recent CCTA study demonstrated that a low-attenuation non-calcified coronary plaque burden was the strongest prognostic marker among other clinical factors such as the presence of CAD and coronary artery calcium score (CACS) [9]. This finding suggests the utility of CCTA for identifying high-risk patients. In a subanalysis of a large clinical trial of patients with chest pain and distinct metabolic phenotypes who underwent CCTA, Kammerlander *et al*. [4] demonstrated that metabolically unhealthy individuals without obesity were at a high risk of ASCVD events. Although DM plays a key role in the pathophysiology of MetS [10], its association with high-risk plaque volume detected by CCTA and cardiovascular consequences remains unclear. This study aimed to investigate the associations among DM, high-risk plaque volume, and cardiovascular outcomes across metabolic phenotypes in patients with suspected CAD who underwent CCTA.

## 2. Methods

### 2.1 Study Population and Metabolic Phenotypes

**Fig. 1.**
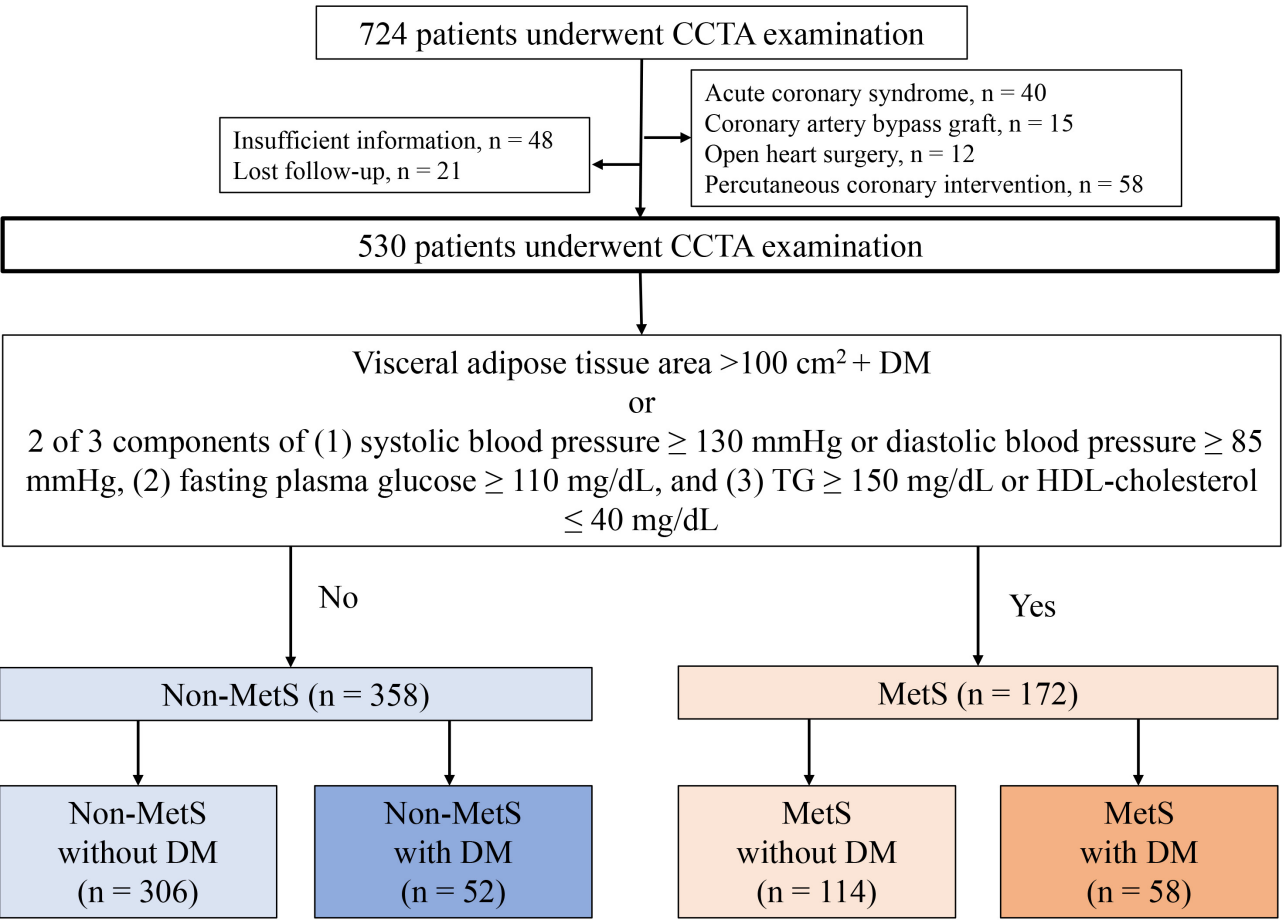
**Flow chart of study patients**. A flowchart illustrating the 530 patients who underwent CCTA examination and their categorisation into non-MetS and MetS groups. Patients were further categorised into four groups according to the presence or absence of metabolic equivalents of MetS and DM. CCTA, coronary computed tomography angiography; DM, diabetes mellitus; MetS, metabolic syndrome; TG, triglyceride; HDL, high-density lipoprotein.

The Institutional Review Board approved the pooled data analysis (no. 2021-A). All participants provided written informed consent for the use of de-identified data including clinical information, laboratory test results, and CCTA imaging results. This retrospective observational study included patients who underwent CCTA between January 2018 and December 2020, were clinically identified as having stable chest symptoms and underwent abdominal computed tomography (CT) to assess abdominal obesity. The exclusion criteria were as follows: (1) diagnosis of acute coronary syndrome; (2) history of coronary artery bypass graft or open-heart surgery; (3) congestive heart failure; (4) history of percutaneous coronary intervention; (5) insufficient patient information; and (6) loss to follow-up. Fig. 1 shows a flowchart of the study population comprising 530 patients.

Non-contrast-enhanced abdominal CT was performed before CCTA to quantify areas with visceral adipose tissue (VAT). The VAT areas were measured at the L2–L3 level using SYNAPSE VINCENT (Fujifilm, Inc., Tokyo, Japan). Abdominal obesity was defined as a VAT area ≥100 ${\mathrm{cm}}^{2}$, corresponding to an abdominal circumference ≥85 cm for men and ≥90 cm for women [11]. MetS was defined according to the Japanese Committee for the Diagnostic Criteria of Metabolic Syndrome [12] as abdominal obesity in the presence of DM (MetS with DM) or two or more of the following components in the absence of DM: (1) systolic blood pressure (BP) ≥130 mmHg or diastolic BP ≥85 mmHg; (2) fasting plasma glucose ≥110 mg/dL; and (3) triglycerides (TGs) ≥150 mg/dL or high-density lipoprotein cholesterol ≤40 mg/dL. In addition, medical therapy using antihypertensive, antidiabetic, and lipid-lowering drugs is considered a component of MetS. Patients were further categorised into four groups according to the presence or absence of metabolic equivalents (MetS) and DM. DM was defined according to the Japanese Clinical Practice Guidelines for Diabetes, 2019 (reference). DM was diagnosed in individuals meeting any of the following criteria: (1) an elevated fasting plasma glucose level of ≥126 mg/dL or casual plasma glucose level of ≥200 mg/dL on at least two different visits; (2) hemoglobin A1c (HbA1c) level of ≥6.5% and either fasting blood glucose level of ≥126 mg/dL or casual blood glucose level of ≥200 mg/dL; or (3) a history of a prior diagnosis, or the need for antidiabetic medication [13].

Pharmacological treatment and lifestyle modifications were recommended for all patients, according to guidelines for hypertension, dyslipidemia, and diabetes [14]. In addition, patients subsequently underwent invasive coronary angiography or coronary revascularization based on the results of CCTA and noninvasive stress tests [15]. According to the Japanese Atherothrombosis Society guidelines, the treatment targets for low-density lipoprotein cholesterol for the primary prevention of ASCVD are <100 mg/dL for high-risk patients, 120 mg/dL for moderate-risk patients, and 140 mg/dL for low-risk patients [14]. The treatment target for fasting TG level was <150 mg/dL. For patients with hypertension, the treatment target for blood pressure was <140/90 mmHg. Particularly for patients with diabetes or chronic kidney disease with albuminuria, the treatment target was <130/80 mmHg [16]. The target glycaemic control for most patients was HbA1c <7.0% to prevent diabetic complications. However, for those experiencing difficulties in glycaemic control owing to hypoglycaemia, a target of <8.0% was considered [13].

### 2.2 CCTA Imaging and Analysis

CCTA was performed using a 320-row multidetector CT scanner (Aquilion ONE/NATURE Edition; Canon Medical Systems Inc., Tokyo, Japan) [17]. A β-blocker and nitrates were given to control heart rate <60 beat per minutes. The scan parameters included a detector collimation of 0.5 × 320 mm, gantry rotation time of 350 ms, tube voltage of 120 kV, and tube current of 130–600 mA. An electrocardiogram-triggered prospective gating method was used for CCTA. The CACS was evaluated using the Agatston method at a fixed thickness of 3 mm [18]. The images were reconstructed using a forward-projected model-based iterative reconstruction solution for coronary artery analysis with a cross-sectional thickness of 0.5 mm and a reconstruction increment of 0.25 mm.

The Agatston scores were categorised as 0, 1–100, 101–400, and >400 Agatston units using SYNAPSE VINCENT version 4.6 (Fujifilm Inc., Tokyo, Japan). Coronary artery diameter stenosis was reported based on a 16-segment American Heart Association model by two observers (K.O. and H.I.). Obstructive CAD was defined as the presence of coronary plaques with a stenosis diameter ≥50% in one or more major epicardial vessels, and/or 50% in the left main coronary segment. Non-obstructive CAD was defined as diameter stenosis of <50% in the epicardial coronary arteries. Patients who did not fall into either category were diagnosed with CAD.

For coronary plaque analysis, coronary artery centerlines were identified semi-automatically; the proximal and distal portions of the coronary plaque lesions were manually defined; and the vessel wall, lumen, and plaque components were auto-segmented and manually adjusted. Based on their composition, lesions were categorised into calcified plaques (CPs) (>150 Hounsfield units [HU]) and non-CPs (NCPs) (<150 HU). A low-attenuation plaque (LAP) was defined as a region with a CT value <30 HU [17]. The percentage of the plaque volume for each component was calculated as the plaque volume divided by the vessel volume. The napkin-ring sign was defined as a ring-like peripheral higher attenuation (no >130 Hounsfield units) with low central CT attenuation [8, 19]. Spotty calcification was defined as the presence of calcified plaque with a diameter <3 mm in any direction, a calcium length less than 1.5× the vessel diameter, and the width of the calcification less than two-thirds of the vessel diameter [19, 20]. The presence of high-risk plaque signatures has been previously reported at the patient level.

Epicardial adipose tissue (EAT) volume was measured from contrast-enhanced CT images using SYNAPSE VINCENT [17]. Several equidistant axial planes were extracted based on the heart size. The upper limit of the slice was set at the bifurcation of the pulmonary artery trunk, whereas the lower limit was set at the last slice that contained any heart structure. In each plane, the software auto-detected a smooth, closed pericardial contour as the region of interest; adipose tissue was identified with CT attenuation values ranging from –250 to –30 HU within the pericardial sac [17]. Finally, EAT volume was calculated as the sum of the EAT areas in each slice. The mean CT value within the measured EAT volume has been reported previously.

### 2.3 Endpoints

The primary endpoint was a major adverse cardiovascular event (MACE), a composite of cardiovascular death, nonfatal myocardial infarction, unstable angina, and symptom- or ischaemia-driven coronary revascularisation. Cardiovascular death was defined as death resulting from cardiovascular causes, including myocardial infarction, sudden cardiac arrest, heart failure, and stroke [21]. Non-fatal myocardial infarction was defined as typical persistent chest pain with elevated cardiac enzyme levels [21]. Unstable angina was defined as new-onset angina, angina exacerbation with light exertion, or angina at rest without elevated cardiac enzyme levels. Symptom- or ischaemia-driven coronary revascularization was defined as coronary revascularization >3 months after CCTA at baseline, with positive functional tests, or with ≥90% diameter stenosis with symptoms.

### 2.4 Statistical Analysis

All statistical analyses were performed using SPSS version 24 software (IBM Corp., Armonk, NY, USA). Categorical variables were presented as absolute and relative frequencies, and continuous variables were presented as mean ± standard deviation. Subject characteristics were compared using a one-way analysis of variance for numerical variables. Categorical variables were analysed using the chi-square or Fisher’s exact tests. Multivariate Cox proportional hazards analysis was used to estimate hazard ratios (HRs) with 95% confidence intervals (CIs). To test the hypothesis that DM or MetS function as predictors of MACEs independent of high-risk plaque volume, DM, MetS, and LAP volume >4% were entered into multivariate Cox proportional hazards models adjusted for the Suita CVD risk score (models 1 and 2). Furthermore, to test the hypothesis that each metabolic phenotype acts as a predictor of MACE development, independent of high-risk plaque volume, each metabolic phenotype, as compared with non-DM without MetS and LAP volume >4%, was entered into the multivariate Cox proportional hazards models adjusted for the Suita CVD risk score (models 3, 4, and 5). Kaplan–Meier curves and log-rank tests were used to depict and assess the differences in cumulative event rates between the groups. Analyses were initiated at the time of CCTA and terminated at the earliest occurrence of the primary endpoint or at the median follow-up (2.91 years). Analyses were censored at the last follow-up or composite events, whichever occurred earlier. Statistical significance was set at *p* < 0.05 (two-sided).

## 3. Results

The mean age of the patients was 64 ± 14 years, and 299 (56%) were men. Baseline characteristics stratified by metabolic phenotype are presented in Table 1. MetS was present in 172 of the 530 patients (32%), and DM was observed in 110 (21%). Of these, DM was observed in 52 patients in the non-MetS (15%) and 58 in the MetS (44%) groups.

**Table 1.** **Patient characteristics according to metabolic phenotypes**.

		Non-MetS		MetS		*p* value
		DM (–) (n = 306)	DM (+) (n = 52)	DM (–) (n = 114)	DM (+) (n = 58)	
Age		64 (15)	69 (10)	62 (13)	66 (12)	<0.001
Male, n (%)		158 (52%)	32 (62%)	70 (61%)	39 (67%)	0.062
BMI, kg/${\mathrm{mm}}^{2}$		22.7 (3.4)	22.1 (2.8)	26.6 (4.2)	27.7 (4.6)	<0.001
VAT area, ${\mathrm{cm}}^{2}$		78 (41)	66 (32)	148 (45)	168 (62)	<0.001
Subcutaneous fat area, ${\mathrm{cm}}^{2}$		137 (70)	105 (67)	200 (98)	192 (82)	<0.001
Systolic BP, mmHg		136 (21)	145 (22)	148 (24)	148 (25)	<0.001
Triglyceride, mg/dL		134 (141)	125 (98)	221 (244)	241 (488)	0.001
HDL-C, mg/dL		67 (19)	65 (17)	56 (15)	50 (13)	<0.001
LDL-C, mg/dL		124 (33)	110 (38)	135 (36)	115 (32)	<0.001
Haemoglobin A1c, %		5.6 (0.3)	7.1 (1.7)	5.7 (0.3)	7.2 (1.8)	<0.001
CRP (LogCRP), mg/L		0.31 (0.86)	0.20 (0.34)	0.31 (0.71)	0.35 (0.49)	<0.001
Hypertension		168 (55%)	44 (85%)	107 (94%)	54 (93%)	<0.001
Diabetes mellitus		0 (0%)	52 (100%)	0 (%)	58 (100%)	-
Dyslipidaemia		155 (51%)	36 (69%)	113 (99%)	49 (84%)	<0.001
Current or former tobacco user		38 (12%)	10 (19%)	21 (18%)	10 (17%)	0.306
CKD		72 (24%)	14 (27%)	29 (25%)	16 (28%)	0.883
Suita CVD risk score		23.5 (10.8)	35.5 (9.3)	26.0 (9.0)	33.9 (9.0)	<0.001
Atrial fibrillation		27 (8.8%)	10 (19%)	9 (7.8%)	9 (16%)	0.056
Medications						
	ACE inhibitor or ARB	47 (15%)	15 (29%)	34 (30%)	21 (36%)	<0.001
	Calcium channel blocker	61 (20%)	18 (35%)	39 (34%)	21 (36%)	0.002
	β-blocker	11 (3.5%)	5 (9.6%)	10 (8.8%)	4 (6.8%)	0.102
	Statins	39 (13%)	18 (35%)	42 (37%)	23 (40%)	<0.001
	Insulin	0 (0%)	4 (7.7%)	0 (0%)	5 (8.6%)	-

Values are given as means ± standard deviations or numbers (%). ACE, angiotensin converting enzyme; ARB, angiotensin Ⅱ receptor blocker; BMI, body mass index; BP, blood pressure; CKD, chronic kidney disease; CRP, C-reactive protein; CVD, cardiovascular disease; VAT, visceral adipose tissue; HDL-C, high-density lipoprotein cholesterol; LDL-C, low-density lipoprotein cholesterol; DM (–), without diabetes mellitus; DM (+), with diabetes mellitus; MetS, metabolic syndrome.

Table 2 shows the baseline CCTA findings of the four groups. The prevalence of a CACS >400 was most significant in the MetS with DM (22%) and non-MetS with DM (21%, overall *p* < 0.001) groups. The frequency of obstructive CAD was highest in the non-MetS with DM group (58%), followed by the MetS with DM (46%), MetS without DM (39%), and non-MetS without DM (36%) groups. The MetS with DM group had greater NCP, LAP, and CP volumes than the MetS without DM group. For the analysis of plaque vulnerability, there was no significant difference in the prevalence of the napkin-ring sign and spotty calcification among the four groups, whereas the MetS with DM group more frequently had a LAP volume of 4% than the other groups (*p* = 0.008). Although significantly greater EAT volumes were observed in individuals with MetS than in those without MetS, there were no significant differences among those with DM. The non-MetS with DM group had the lowest mean CT value of the EAT among the four groups (*p* < 0.001).

**Table 2.** **CCTA findings according to metabolic phenotypes**.

		Non-MetS		MetS		*p* value
		DM (–) (n = 306)	DM (+) (n = 52)	DM (–) (n = 114)	DM (+) (n = 58)	
CACS						
	CACS 0	153 (50%)	13 (25%)	49 (43%)	14 (24%)	<0.001
	CACS 1–100	77 (25%)	13 (25%)	36 (32%)	14 (24%)	0.569
	CACS 101–400	54 (18%)	15 (29%)	19 (17%)	17 (29%)	0.056
	CACS >400	22 (7.2%)	11 (21%)	10 (8.8%)	13 (22%)	<0.001
Stenosis severity on CCTA						
	No CAD	64 (21%)	5 (9.6%)	19 (17%)	5 (8.6%)	0.048
	Non-obstructive CAD	131 (43%)	17 (33%)	51 (45%)	26 (45%)	0.490
	Obstructive CAD	111 (36%)	30 (58%)	44 (39%)	27 (46%)	0.021
Coronary plaque burden and composition						
	NCP volume, %	19.7 (5.7)	20.6 (6.6)	21.2 (6.9)	23.4 (8.4)	<0.001
	LAP volume, %	2.4 (1.7)	3.2 (2.6)	3.3 (2.9)	4.4 (5.0)	0.001
	CP volume, %	0.7 (2.4)	2.1 (3.9)	0.9 (2.9)	1.6 (3.7)	0.002
	LAP volume >4%, n (%)	50 (16%)	12 (23%)	29 (25%)	20 (34%)	0.008
	Napkin-ring sign, n (%)	54 (18%)	14 (27%)	27 (24%)	12 (21%)	0.313
	Spotty calcification, n (%)	99 (32%)	14 (27%)	35 (31%)	18 (31%)	0.887
	EAT volume, mL	105 (42)	107 (40)	148 (46)	176 (46)	<0.001
	EAT mean CT value, HU	–79.0 (5.3)	–77.7 (5.3)	–80.7 (4.0)	–79.5 (5.0)	0.001

Values are given as means ± standard deviations or numbers (%). CACS, coronary artery calcium score; CAD, coronary artery disease; CP, calcified plaque; DM (–), without diabetes mellitus; DM (+), with diabetes mellitus; EAT, epicardial adipose tissue; MetS, metabolic syndrome; NCP, non-calcified plaque; LAP, low-attenuation plaque; CCTA, coronary computed tomography angiography; CT, computed tomography; HU, Hounsfield units.

### Primary Outcome

**Fig. 2.**
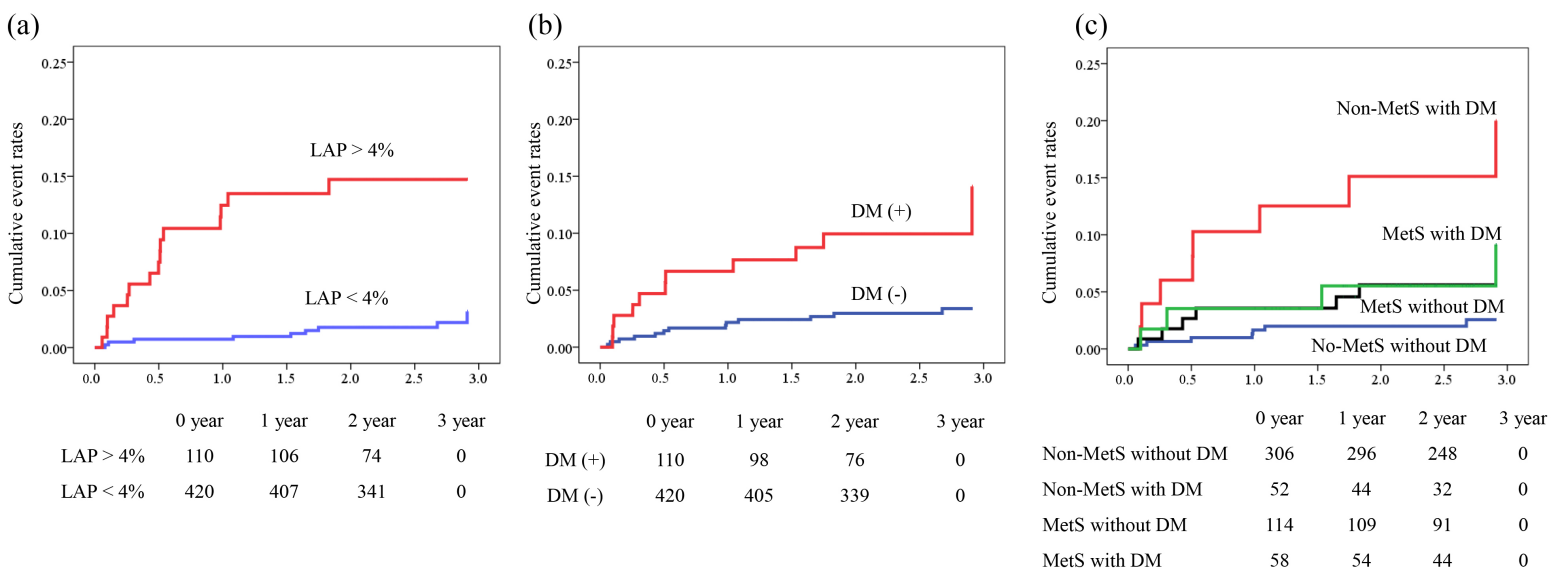
**Kaplan–Meier analysis for prediction of MACEs**. Kaplan–Meier curves demonstrating significant differences in cumulative event rates between metabolic phenotypes using a composite endpoint of cardiovascular death, acute coronary syndrome, and symptom- or ischaemia-driven coronary revascularization. (a) Patients with and without LAP volume >4%; (b) patients with and without DM; and (c) higher event rates in non-MetS patients with DM, followed by those with MetS and DM. LAP, low-attenuation plaque; DM, diabetes mellitus; MetS, metabolic syndrome; MACE, major adverse cardiovascular events; DM (–), without diabetes mellitus; DM (+), with diabetes mellitus.

During a mean follow-up period of 2.7 ± 0.9 years (median 2.91 years), MACEs were observed in 25 patients (4.7%). Table 3 summarises the unadjusted Cox proportional hazard models used to predict the primary endpoints. CACS >400 (*p* < 0.001), napkin-ring sign (*p* < 0.001), LAP volume >4% (*p* < 0.001), obstructive CAD (*p* < 0.001), and DM (*p* < 0.001) were significantly associated with the primary endpoint. In the multivariable Cox proportional hazards model analysis (Table 4), DM as a predictor was associated with the primary endpoint, independent of LAP volume >4% (HR for DM in model 1; HR, 2.68; 95% CI, 1.16–6.18; *p* = 0.02), although MetS did not function as an independent predictor (model 2 in Table 4). Fig. 2 illustrates the Kaplan–Meier curve analysis stratified by the presence or absence of LAP >4% (Fig. 2a) and DM (Fig. 2b). Patients with DM and LAP >4% had a higher incidence of MACEs than those without LAP (both *p* < 0.001, log-rank test). In the subgroup analysis of DM (n = 110), Spearman’s correlation test demonstrated that %LAP volume was not correlated with HbA1c level (ρ= 0.12, *p* = 0.31), while %LAP volume >4% tended to be associated with MACE (HR, 2.72; 95% CI, 0.875–8.43; *p* = 0.084).

**Table 3.** **Unadjusted Cox proportional hazards model for the prediction of primary outcomes**.

	Non-adjusted HR	95% CI	*p* value
All patients (n = 530)			
Age	1.03	1.002–1.07	0.040
Male	1.65	0.72–3.88	0.229
CACS >400	5.09	2.25–11.54	<0.001
LAP volume >4%	6.03	2.70–13.40	<0.001
Napkin-ring sign	5.43	2.46–11.97	<0.001
Spotty calcification	1.24	0.55–2.81	0.602
Obstructive CAD	8.45	2.90–24.60	<0.001
EAT volume	1.001	0.99–1.01	0.853
EAT mean CT value	1.03	0.99–1.08	0.126
BMI ≥25 kg/$m^{2}$	0.45	0.16–1.20	0.111
VAT ≥100 ${\mathrm{cm}}^{2}$	1.28	0.58–2.80	0.535
MetS	1.39	0.62–3.10	0.415
DM	3.74	1.70–8.20	0.001
Chronic kidney disease	1.64	0.80–3.39	0.175
CRP	1.25	0.70–2.23	0.449

CRP was log-transformed for analysis. BMI, body mass index; CACS, coronary artery calcium score; CAD, coronary artery disease; CI, confidence interval; CRP, C-reactive protein; EAT, epicardial adipose tissue; HR, hazard ratio; VAT, visceral adipose tissue; DM, diabetes mellitus; MetS, metabolic syndrome; CT, computed tomography; LAP, low-attenuation plaque.

**Table 4.** **Cox proportional hazards analysis for the prediction of major cardiovascular adverse events**.

	Predictor	HR	95% CI	*p* value
Model 1	DM (Reference, non-DM)	2.68	1.16–6.18	0.02
	Low-attenuation plaque volume >4%	5.41	2.42–12.11	<0.001
Model 2	MetS (Reference, non-MetS)	0.99	0.44–2.23	0.99
	Low-attenuation plaque volume >4%	5.83	2.59–13.10	<0.001
Model 3	DM without MetS (Reference, non-DM without MetS)	6.89	2.33–20.39	0.001
	Low-attenuation plaque volume >4%	9.66	3.29–28.35	<0.001
Model 4	DM with MetS (Reference, non-DM without MetS)	1.69	0.56–5.09	0.34
	Low-attenuation plaque volume >4%	9.24	2.82–30.27	<0.001
Model 5	Non-DM without MetS (Reference, non-DM without MetS)	2.24	0.58–8.53	0.23
	Low-attenuation plaque volume >4%	4.62	1.37–15.51	0.013

Models 1–5 were adjusted for the Suita CVD risk score. CI, confidence interval; DM, diabetes mellitus; HR, hazard ratio; MetS, metabolic syndrome; CVD, cardiovascular disease.

In the subgroup analysis of each metabolic phenotype (non-DM without MetS as a reference), DM without MetS functioned as a predictor of MACE independent of LAP volume >4% (model 3), whereas DM with MetS (model 4) or non-DM with MetS (model 5) did not reach statistical significance. The incidence rate of the composite endpoint was the highest in the non-MetS with DM group, followed hierarchically by the MetS with DM, MetS without DM, and non-MetS without DM groups (*p* < 0.001, log-rank test; Fig. 2c).

## 4. Discussion

This study investigated the association between distinct metabolic phenotypes, defined by the presence or absence of MetS and DM, and cardiovascular outcomes in symptomatic patients who underwent CCTA. The key findings of this study were as follows: (1) among the four groups, the most unfavourable prognosis was observed in patients without MetS but with DM, compared to those with MetS with or without DM; (2) a LAP volume >4% was identified as a robust predictor of MACE across different metabolic phenotypes; and (3) DM, independent of LAP volume >4%, was a predictor of MACE, whereas MetS did not show a significant predictive value.

### 4.1 Metabolic Disorders, High-risk Plaque Burden, and Outcomes

Several large clinical trials have demonstrated that myocardial ischaemia is an important surrogate marker for improving outcomes in patients with stable CAD, whereas ischaemia-guided management has a limited ability to prevent acute coronary events compared to optimised medical therapy [22, 23, 24]. These findings raise questions about the credibility of ischaemia-guided management of patients with stable CAD, redirecting attention toward coronary microvascular dysfunction and high-risk plaque burden [24]. An increased plaque burden, especially of noncalcified plaques, has been reported in patients with MetS [25]. Yonetsu *et al*. [25] used optical coherence tomography to demonstrate that MetS is associated with an increased burden of lipid-rich plaques. Although these findings indicate a potential link between obesity, metabolic disorders, and unfavourable coronary plaque features, there is limited knowledge regarding coronary plaque burden in distinct metabolic phenotypes with and without DM.

Previous clinical studies have reported an inverse association between BMI and cardiovascular prognosis (obesity paradox) [3, 4, 5]. In patients with ASCVD and DM, Pagidipati *et al*. [5] demonstrated that overweight or obese individuals had a lower cardiovascular risk than those with normal weight. In a subanalysis of a large clinical trial of patients with chest pain who underwent CCTA, Kammerlander *et al*. [4] demonstrated that of all patients with distinct metabolic phenotypes, metabolically unhealthy individuals without obesity exhibited a significantly high risk of plaque burden and ASCVD events. This paradoxically benign effect of obesity may be explained by its protective effect against atherosclerosis. Although obesity can cause inflammation in perivascular adipose tissues and exacerbate atherosclerotic lesion formation, adipose tissue plays a role in atheroprotection under healthy conditions [26]. Clinically, obesity may be associated with metabolic reserves in older patients by protecting against malnutrition, frailty, and osteoporosis [3]. Patients with obesity lack sarcopenia and have limited exercise capacity and reduced mobility, which are associated with increased cognitive decline, heart failure, and mortality. This might explain our observation that patients with MetS had better cardiovascular outcomes, albeit with a high prevalence of LAP (4%).

In addition, increased fasting plasma glucose levels were observed in patients without non-MetS DM. Hyperglycaemia and insulin resistance have been reported to be key drivers of calcification in DM [10, 27]. Liu *et al*. [28] showed that higher glucose levels and their variability are associated with plaque rupture in patients with ST-segment elevation myocardial infarction. In line with these observations, we found that a LAP ≥4% was a robust predictor of cardiovascular events across distinct metabolic phenotypes and tended to be associated with DM. These findings suggest that metabolic phenotypes can help identify patients at a high risk of cardiovascular events, in addition to a high-risk plaque burden.

### 4.2 EAT, Plaque Characteristics, and Outcomes

CAD is a chronic inflammatory disease associated with the underlying risk of metabolic disorders [29]. A close relationship has been reported between abdominal visceral obesity and increased coronary atherosclerotic burden [7, 30]. Our results demonstrated that MetS patients with or without DM had increased EAT and LAP volumes, whereas non-MetS patients with DM had the worst outcomes with lower EAT and LAP volumes, indicating an alternating pathophysiology of acute coronary syndrome. Our findings are consistent with those of Kammerlander *et al*. [4], who demonstrated that both metabolically unhealthy obese and non-obese patients exhibit increased high-risk plaques. The increased prevalence of obstructive CAD and vascular CP in DM patients may explain this finding [31]. Distinct plaque characteristics may reflect the different stages (advanced or less advanced) of coronary atherosclerosis, resulting in different responses to lipid-lowering therapy [32]. Furthermore, previous studies investigating plaque structural stress have demonstrated that microcalcifications contribute to increased stress, leading to plaque rupture and myocardial infarction [33]. These observations provide insight into the poorer outcomes observed in DM patients without MetS in this study.

Although we observed that the EAT volume was not correlated with cardiovascular outcomes, the mean CT value of the EAT was (Table 3). EAT has been associated with coronary atherosclerosis, calcification, and cardiovascular outcomes, and has attracted attention as a therapeutic target [34, 35, 36]. This association has motivated the development of imaging methods that enable the assessment of inflammation in pericoronary adipose tissue, which interacts with the underlying vascular wall by producing proinflammatory adipokines [37]. In a retrospective CCTA study, Oikonomou *et al*. [38] demonstrated that an increased fat attenuation index (FAI) around the epicardial coronary arteries predicted cardiovascular outcomes. Moreover, a recent meta-analysis demonstrated that higher pericoronary FAI values offer additional prognostic value for MACE in 6335 patients analyzed in prospective follow-up clinical studies [39]. A higher CT attenuation of the EAT indicates an increased inflammatory status, which supports our finding that non-MetS patients with DM had the worst prognosis. Noninvasive assessment of coronary plaque burden and metabolic phenotypes allows for further risk stratification of symptomatic patients undergoing CCTA.

### 4.3 Study Limitation

This study included a relatively small number of patients, and event rates during follow-up were relatively low (<5%). Our findings should be interpreted with caution because the patients in the DM with MetS group were significantly older than those in the other groups with more obstructive CAD. However, further studies are required to confirm these findings. Furthermore, although a unified definition is required, the criteria for abdominal obesity vary according to race [40]. In this study, we used the quantitative VAT values obtained from CT scans to define abdominal obesity. In our institution, we employed the definition for adipose tissue as attenuation values ranging from –250 to –30 HU. The attenuation values ranging from –190 to –30 HU would be the most common definition to measure adipose tissues [39]. There were no statistically significant differences between the two methods in the selected consecutive 30 patients. Additionally, this study lacked information on the duration of DM. Although the duration of DM may affect plaque vulnerability and clinical event rates [41], we did not analyse the association between DM duration and outcomes. Lastly, we did not perform laboratory tests, such as HOMA-IR, to measure insulin resistance, which is associated with inflammation [42] and plaque vulnerability [10]. Further studies are needed to investigate the association between insulin resistance and MACE in this population.

## 5. Conclusions

Individuals with DM (without MetS) had a significantly higher risk of developing MACEs than those with MetS. This observation indicates that DM is an independent predictor of ASCVD events, regardless of the presence of obstructive CAD or high-risk plaque volume.

## 6. Clinical Perspective

This study investigated the association among DM, high-risk coronary plaque burden, and MACEs across metabolic phenotypes stratified by the presence or absence of MetS and DM in patients with suspected CAD who underwent CCTA.

Among the four metabolic phenotypes, the incidence of MACEs was the highest in the non-MetS with DM group, followed hierarchically by the MetS with DM, MetS without DM, and non-MetS without DM groups. A LAP volume of >4% is a robust predictor of MACEs among metabolic phenotypes. Furthermore, DM, independent of a LAP volume >4%, was a predictor of MACEs.

## Data Availability

The data that support the findings of this study are available from the corresponding author on reasonable response.

## Abbreviations

ACS, acute coronary syndrome; ASCVD, atherosclerotic cardiovascular disease; CACS, coronary artery calcium score; CAD, coronary artery disease; CCTA, coronary computed tomography angiography; CP, calcified plaques; DM, diabetes mellitus; LAP, low-attenuation plaque; MACE, major adverse cardiovascular events; MetS, metabolic syndrome; NCP, noncalcified plaque.
